# Monocyte chemoattractant protein (*MCP*)-1 *−*2518 A/G SNP in Chinese Han patients with VKH syndrome

**Published:** 2009-08-08

**Authors:** Shengping Hou, Peizeng Yang, Lin Xie, Liping Du, Hongyan Zhou, Zhengxuan Jiang

**Affiliations:** 1 The First Affiliated Hospital, Chongqing Medical University, Chongqing Key Laboratory of Ophthalmology, and Chongqing Eye Institute, Chongqing, P.R. China; 2 Department of Ophthalmology, Daping Hospital, the Third Military Medical University, Chongqing, P.R. China; 3 Uveitis Study Center, Sun Yat-sen University, Guangzhou, P.R. China

## Abstract

**Purpose:**

Vogt-Koyanagi-Harada (VKH) syndrome is an autoimmune disease. The monocyte chemoattractant protein-1 (*MCP-1*) gene has been implicated in the pathogenesis of certain autoimmune diseases. The aim of this study was to examine whether a *MCP-1* polymorphism was associated with VKH syndrome.

**Methods:**

A case-control analysis was performed using genomic DNA samples from 307 VKH patients and 319 age-, sex-, and ethnically-matched healthy controls. The *MCP-1* polymorphism at the −2518 A/G locus was genotyped using polymerase chain reaction restriction fragment length polymorphism (PCR-RFLP) assay.

**Results:**

The distribution of genotypic frequency of the *MCP-1* −2518 A/G polymorphism in all subjects did not deviate from Hardy–Weinberg equilibrium (HWE; p>0.05). Allelic and genotypic frequency analysis revealed no significant difference between VKH patients and healthy controls for the *MCP-1* −2518 A/G polymorphism (p>0.05). No significant differences were   found according to gender and neither was found according to extraocular findings including neck stiffness, tinnitus, alopecia, poliosis, dysacusia, scalp hypersensitivity, and vitiligo.

**Conclusions:**

The result suggests that the susceptibility to VKH syndrome in Chinese Han patients may be not influenced by the *MCP-1* −2518 A/G polymorphism.

## Introduction

Vogt-Koyanagi-Harada (VKH) syndrome is a multisystem disorder mainly affecting pigmented tissues in the eye, auditory, integumentary, and central nervous systems. The clinical features of VKH syndrome include bilateral granulomatous panuveitis frequently associated with extraocular findings including pleocytosis in the cerebrospinal fluid (CSF), dysacusis, alopecia, poliosis, and vitiligo [1-3]. Although the etiology and pathogenesis of VKH syndrome remain unclear, numerous studies have suggested that this syndrome is considered to be a cell-mediated autoimmune disease directed against melanocytes. Epidemiological studies show that VKH syndrome is more prevalent in certain racial and ethnic groups, particularly in pigmented groups [3] such as Asian and Latin-American populations and displays an adventive familial aggregation pattern [4]. Two human leukocyte antigen (*HLA*) genes, *HLA*-*DR4* and *HLA-DRw53*, have been shown to be associated with VKH patients in various ethnic groups including the Chinese and Japanese [5-7]. These evidences suggest that immune-associated genetic factors may play a crucial role in the pathogenesis of VKH syndrome. However, the previously reported genetic risks for VKH syndrome such as *HLA-DR4* and *HLA-DRw53* account for only a small portion of the overall estimated risk for this syndrome, suggesting that non-*HLA* genes may confer a substantial proportion of genetic susceptibility to this syndrome. Therefore, studies on the disease association with genes involved in the immune response may highlight the genetic susceptibility to VKH syndrome.

*MCP-1* is a potent chemokine released by lymphocyte, monocytes, mast cells, and eosinophils during inflammation and also produced by ocular cells such as the retinal pigment epithelial cell cultured in vitro [8]. *MCP-1* has been implicated in the recruitment of leukocytes into the site of inflammation. The elevated *MCP-1* concentration in the aqueous humor of active anterior uveitis patients [9] and ocular fluids and tissues during ocular inflammation [9-13] combined with the recent reports showing an association of *MCP-1* −2518 A/G polymorphism with uveitis [14-16] and multiple autoimmune diseases [17-19] suggest that *MCP-1* may be involved in the pathogenesis of VKH syndrome. The present study was therefore designed to examine the association of the *MCP-1* −2518 A/G polymorphism with VKH syndrome using a case–control association study.

## Methods

### Subjects

This study was approved by the ethics committees of our hospitals and adhered to the tenets of the Declaration of Helsinki. Additionally, informed consent was obtained from each participant or each participant’s guardian. After giving informed consent, blood samples were collected from 307 Chinese Han VKH patients and 319 age- and sex-matched, unrelated, healthy Chinese Han controls, which were recruited from Zhongshan Ophthalmic Center, Sun Yat-sen University (Guangzhou, P.R. China) and the First Affiliated Hospital, Chongqing Medical University (Chongqing, P.R. China) from April 2005 to March 2009. All VKH patients were diagnosed according to the revised criteria for VKH syndrome [20].

### DNA extraction

Genomic DNA was prepared from peripheral blood mononuclear cells (PBMCs) of VKH patients and healthy controls using the QIAamp DNA Mini Blood Kit (Qiagen, Hilden, Germany) according to manufacturer’s instructions. DNA samples were collected in a 1.5 ml Eppendorf tube and stored at −20 °C until use.

### Genotyping

Genotyping for the *MCP*-*1* polymorphism (−2518A/G, rs1024611 in dbSNP database) was performed using polymerase chain reaction restriction fragment length polymorphism (PCR-RFLP). The forward primer, 5′-CCG CAT TCA ATT TCC CTT TAT-3′, and reverse primer, 5′-TTC CAA AGC TGC CTC CTC A-3′, were designed using primer premier 5.0 software (Premier Biosoft International, Palo Alto, CA). PCR reactions were performed under the following conditions: 10 min at 94 °C followed by 35 cycles at 95 °C for 45 s, 55 °C for 45 s, 72 °C for 45 s, and then for 3 min at 72 °C. The PCR products were incubated with PvuII at 37 °C (New England Biolabs Inc., Ontario, Canada) for at least 3 h and separated on 2% agarose gels. Ten percent of the PCR samples were directly sequenced to confirm the polymerase chain reaction restriction fragment length polymorphism (PCR-RFLP) results (Invitrogen Biotechnology Co., Shanghai, P.R. China).

### Statistical analysis

Statistical analysis was performed with the SPSS version 12.0 for Windows (SPSS Inc., Chicago, IL). Hardy–Weinberg Equilibrium (HWE) was tested by the χ^2^ test. We evaluated the frequency of genotypes and alleles in this study using the χ^2^ test. All statistical tests were two-sided, and statistical significance was taken when p<0.05.

## Results

Clinical findings of VKH patients and characteristics of controls are presented in Table 1. The mean age of VKH patients was 34.3±10.3 years and that of healthy controls was 34.7±11.6 years (Table 1). There was no significant difference in the distribution of age between VKH patients and controls (p>0.05).

**Table 1:** Clinical findings of patients with VKH syndrome.

**Clinical findings**	**VKH Patients**	
	**Total (n=307)**	**%**
Age at onset (years±SD)	34.3±10.3	
Male/Female	171/136	
Neck stiffness	136	44.3%
Alopecia	109	35.5%
Poliosis	96	31.3%
Vitiligo	48	15.6%
Dysacusia	73	23.8%
Tinnitus	117	38.1%
Scalp hypersensitivity	48	15.6%

This table summarizes the clinical findings of VKH patients.

Three hundred and seven VKH patients and 319 healthy controls were genotyped for the *MCP-1* −2518 A/G polymorphism. The result of genotyping was matched with that of direct sequencing for 10% of the samples. The distribution of genotypic frequency of this single nucleotide polymorphism (SNP) in all subjects did not show a significant deviation from Hardy–Weinberg equilibrium (HWE; p>0.05).

The distributions of genotypic and allelic frequencies of the *MCP-1* −2518 A/G polymorphism are shown in Table 2. No genetic association was demonstrated between the −2518A/G polymorphism of *MCP-1* and VKH syndrome in an allele and genotype analysis, although the roles of *MCP-1* in the immune diseases suggest its possible involvement in the development of VKH syndrome. In addition, our results did not indicate a significant difference when allelic and genotypic frequencies of the *MCP-1* −2518 A/G polymorphism were analyzed according to gender and any one of extraocular findings including neck stiffness, tinnitus, alopecia, poliosis, dysacusia, scalp hypersensitivity, and vitiligo.

**Table 2:** Frequencies of alleles and genotypes of *MCP-1* polymorphism in VKH Patients and controls.

**SNP**	**Genotype/ Allele**	**VKH (n=307)**	**Controls (n=319)**	**χ^2^**	**p value**	**OR (95% CI)**
*MCP-1* −2518 A/G	AA	76 (24.8%)	89 (27.9%)	0.797	0.372	0.85 (0.60–1.21)
	AG	152 (49.5%)	135 (42.3%)	3.259	0.071	1.34 (0.98–1.83)
	GG	79 (25.7%)	95 (29.8%)	1.277	0.258	0.82 (0.58–1.16)
	A	304 (49.5%)	313 (49.1%)	0.026	0.873	1.02 (0.82–1.27)
	G	310 (50.5%)	325 (50.9%)	0.026	0.873	0.98 (0.79–1.23)

The genotypic and allelic frequencies  of MCP-1 -2518 A/G polymorphism were compared between patients and controls. No significant differences of the genotypic and allelic frequencies were found betweeen VKH patients and controls.

## Discussion

The present study was to investigate the association of the *MCP-1* polymorphism with VKH syndrome in the Chinese Han population. The results showed that there was no association between the *MCP-1* polymorphism and VKH syndrome. The stratification analysis according to gender or extraocular findings did not show any association.

VKH syndrome is a multifactorial autoimmune disease that may result from interactions between susceptibility genes and environmental factors. Several studies have been performed to investigate the genetic risk for VKH syndrome. Recently, we reported two susceptible genes to VKH syndrome including cytotoxic T lymphocyte-associated antigen-4 (*CTLA-4*) and programmed cell death 1 (*PDCD1*) in the Chinese population [21,22]. Horie and coworkers [23,24] showed a lack of association of the tyrosinase gene family (*TYR*, *TYRP1*, and *DCT*) and interferon-γ with VKH syndrome in Japanese patients. In this study, we tested whether the −2518 A/G polymorphism of *MCP-1*, a crucial chemokine, was associated with Chinese Han VKH patients. As a case–control association study may be influenced by various factors including population composition and stratification, the present study drew up the following attempts to ensure the analysis results. First, the controls and patients were strictly matched according to the place where they were born to exclude the possible influence of stratification of the population. Individuals with any autoimmune disease and ocular inflammation were excluded from the controls by careful inquiry of history and, if necessary, by relevant examinations. Second, we restricted our study population to Chinese Han descent to minimize confounding by ethnic variation. Third, the number of donors enrolled in this study (307 VKH patients and 319 age-, sex-, ethnically-matched healthy controls) was large enough to avoid a bias of the results. Finally, direct sequencing for 10% of the samples was performed to validate the result of genotyping.

Several studies have been focused on the association of the *MCP-1* −2518 A/G polymorphism with certain autoimmune diseases [15,25,26]. The investigated polymorphism, which is located in the promoter region of *MCP-1*, has been shown to affect the transcriptional activation of this gene [19]. Therefore, this polymorphism is considered a good candidate in the genetic predisposition to autoimmune diseases. Thus, we focused our study on the association of this polymorphism with VKH syndrome. Unexpectedly, no association was found in these VKH patients. As VKH syndrome is a multisystem disorder characterized by ocular and various extraocular findings, we further performed an association study of the *MCP-1* polymorphism with these extraocular findings. Similarly, we did not find any association of this polymorphism with any one of these clinical findings. This result is consistent with the recent reports, which demonstrated a lack of association of the *MCP-1* −2518 A/G polymorphism with multiple sclerosis (MS) and ulcerative colitis (UC) [27]. Contrary to our observation, this polymorphism was found to be significantly associated with rheumatic arthritis (RA), systemic lupus erythematosus (SLE), and juvenile rheumatoid arthritis (JRA) [25,28,29]. Taken together, these data suggest that the *MCP-1* −2518 A/G polymorphism may play a role in certain but not all autoimmune diseases.

Likewise with other candidate gene studies, several possible limitations exist in our study. A complex multigenic disease involves the interaction of various genetic and environmental factors. Therefore, it is unlikely that any single gene has an exclusive effect on the development of VKH syndrome. The present study only examined the association of the *MCP-1* polymorphism with VKH syndrome but did not refer to the association study of the other susceptible genes. Furthermore, the tested polymorphism in this study is only one of the polymorphisms of *MCP-1*. It is necessary to examine all the tag SNPs of *MCP-1* to understand the precise association of *MCP-1* with VKH syndrome. In addition, the patients enrolled in this study were recruited from Chinese Han individuals, and so the results presented here need to be confirmed using different ethnic populations.

In summary, the present study suggests that there was no association between the *MCP-1* −2518 A/G polymorphism and VKH syndrome. This result remained in stratification analysis according to the gender and various clinical findings.
